# Social status and ejaculate composition in the house mouse

**DOI:** 10.1098/rstb.2020.0083

**Published:** 2020-10-19

**Authors:** Helen L. Bayram, Catarina Franco, Philip Brownridge, Amy J. Claydon, Natalie Koch, Jane L. Hurst, Robert J. Beynon, Paula Stockley

**Affiliations:** 1 Mammalian Behaviour and Evolution Group, University of Liverpool, Leahurst Campus, Chester High Road, Neston CH64 7TE, UK; 2 Centre for Proteome Research, University of Liverpool, Biosciences Building, Crown Street, Liverpool L69 7ZB, UK

**Keywords:** social dominance, sperm competition, ejaculates, seminal fluid, mating plugs, proteomics

## Abstract

Sperm competition theory predicts that males should tailor ejaculates according to their social status. Here, we test this in a model vertebrate, the house mouse (*Mus musculus domesticus*), combining experimental data with a quantitative proteomics analysis of seminal fluid composition. Our analyses reveal that both sperm production and the composition of proteins found in seminal vesicle secretions differ according to social status. Dominant males invested more in ejaculate production overall. Their epididymides contained more sperm than those of subordinate or control males, despite similar testes size between the groups. Dominant males also had larger seminal vesicle glands than subordinate or control males, despite similar body size. However, the seminal vesicle secretions of subordinate males had a significantly higher protein concentration than those of dominant males. Moreover, detailed proteomic analysis revealed subtle but consistent differences in the composition of secreted seminal vesicle proteins according to social status, involving multiple proteins of potential functional significance in sperm competition. These findings have significant implications for understanding the dynamics and outcome of sperm competition, and highlight the importance of social status as a factor influencing both sperm and seminal fluid investment strategies.

This article is part of the theme issue ‘Fifty years of sperm competition’.

## 1. Introduction

Sperm competition [1] is defined as competition between the ejaculates of different males to fertilize a given set of eggs. Following the pioneering theoretical work of Parker [1–5], celebrated in this special issue, sperm competition is widely recognized as a key selective force in the evolution of male ejaculate traits. According to sperm competition theory, males should allocate available resources to ejaculates prudently, according to likely success in sperm competition [4,6]. However, ejaculates consist of a complex mixture of sperm and seminal fluid proteins [7], and while optimal investment strategies for sperm have been the subject of significant theoretical and empirical interest [4,5,8], there are still relatively few tests of predictions for optimal investment in seminal fluid production [9–11]. Nonetheless, there is growing evidence of plasticity in the seminal fluid proteome in relation to sperm competition risk [12–16], and examples suggesting that such plasticity may be modulated by male social status [14,17,18].

In species with a hierarchical social system, dominant males typically have a competitive advantage, placing them in a favoured role during sperm competition [8]. For example, dominant male mammals can often secure greater access to females, allowing them to mate more often, or at an optimal time relative to ovulation [8,19,20]. In this scenario, theoretical models predict that a subordinate male, mating in a disfavoured role, should increase investment in sperm production to compensate for an inherent disadvantage during sperm competition [8,21]. Additionally, it is predicted that males mating in a disfavoured role should increase the allocation of resources to other, non-sperm, components of the ejaculate [9]. While a number of empirical studies support the prediction that subordinate males should invest relatively more into sperm production than dominant males [22,23], the same trend has not previously been demonstrated for rodents. Rather, subordinate male rodents have been found to invest less in sperm production than dominant males [24–27]. However, as yet, it is unknown how male rodents allocate resources among non-sperm ejaculate components according to social status. Hence, it is possible that subordinate males may partly compensate for their disadvantaged role in sperm competition by investing relatively more in the production of functionally relevant non-sperm ejaculate components. For example, in rodents, the seminal fluid proteins are used to produce a substantial copulatory plug, which is thought to promote male success in sperm competition by promoting transport of the mating male's own sperm, and/or blocking the sperm of rival males [28,29]. Subordinate males might, therefore, benefit by investing more in the production of key proteins used in forming these plugs, potentially facilitating the production of plugs that are more difficult for rival males or females to dislodge. Rodent seminal fluid proteins also have known functions in influencing sperm motility and capacitation [30–34], differential investment that could reduce the disadvantage experienced by subordinate males under sperm competition.

The house mouse (*Mus musculus domesticus*) has a complex social system in which dominant males defend territories and are preferred as mates by females [35–38]. Accordingly, dominant males achieve more copulations than subordinates [36] and sire more litters [39]. Female house mice often mate with more than one male, resulting in a moderate level of multiple paternity in natural populations [40]. Subordinate males of laboratory strains produce fewer and less motile sperm than dominant males [24,41]. However, as yet, it is unknown if the seminal fluid proteins of male house mice differ according to their social status.

Here, we test how investment in both sperm and seminal fluid proteins of male house mice differs according to social status. We compare epididymal sperm numbers and reproductive morphology of dominant, subordinate and control (socially isolated) males, and employ a label-free quantitative proteomic approach to quantify differences in their seminal fluid proteins. In addition to analysing those proteins known to be functionally important, this approach allows subtle differences in the expression of all secreted seminal vesicle proteins to be explored.

## 2. Methods

### (a) Subjects

Male house mice were from an outbred colony, founded by wild mice captured in Cheshire, UK. All animals were housed in M3 cages (North Kent Plastic Cages Ltd, UK, 48 cm × 15 cm × 13 cm) on Absorb 10/14 substrate with shredded paper nest material and cardboard enrichment. Food (LabDiet 5002) and water were provided ad libitum*.* Animals were maintained under controlled environmental conditions: temperature 20–21°C, relative humidity 45–65% and a reversed 12 : 12 h light cycle (lights off at 08.00). Subjects were weaned into single-sex sibling groups at age 26 days, and transferred to experimental treatments within 1–2 days.

### (b) Experimental design

A matched-pairs design was used to compare ejaculate traits of sibling males that were housed in sibling pairs to form dominance relationships (dominant versus subordinate). An additional group of males originating from the same litters were singly housed for comparison. This group is hereafter referred to as a control condition, and was intended primarily to provide a comparison with reproductive traits of subordinate males, in order to test for evidence that sperm production or the expression of other reproductive traits are suppressed in the presence of a dominant male. Subjects (*n* = 24) originated from seven different litters. Paired males (*n* = 16) consisted of six pairs, each originating from six different litters, and two pairs originating from one litter. Singly housed males (*n* = 8) originated from five of the same litters as the paired males, and sibling-matched trios were distributed evenly across treatment groups where possible (see electronic supplementary material, dataset). Since not all litters contained sufficient males to achieve a fully matched design, we adopted a different statistical approach to the comparison of paired males, which were always littermates, and the comparison of singly housed and paired males, which were not (see §2e).

To allow individual identification of paired subjects, a small patch of fur was clipped from the hindquarters of one in each pair, with equivalent handling for other subjects. To stimulate normal sexual development [42], males were exposed to soiled bedding from unrelated females every two weeks for the duration of the study. Pairs were observed daily to monitor their behaviour, and were separated to individual cages if necessary to prevent escalated aggression. Five pairs were split, although all but one pair remained together until the final week of the study (separation duration ranged from 3 to 12 days). Dominance relationships were maintained after separation via the daily exchange of sibling pairs between one another's home cages, providing continued exposure to fresh scent of the partner. All pairs were split on the penultimate day of the experiment, so that measurements of reproductive traits could be taken blind to treatment group.

#### (i) Establishing dominance status

Dominant male mice deposit significantly more urinary scent marks than subordinates [43], and these can be visualized using ultraviolet illumination [44]. The scent-marking behaviour of subjects was, therefore, quantified to assess their dominance status. Behaviour was assayed after three weeks in the treatment groups. Scent marking responses of subjects were recorded in response to a standardized competitive stimulus of pooled urine collected from adult male house mice from the same captive colony. Subjects were placed for 45 min in a clean MB1 cage (45 × 28 × 13 cm, North Kent Plastics, UK), lined with Benchkote and marked centrally with 10 µl of stimulus urine. Scent marks were imaged using a UV scanner (InGenuis EPI UV kit and GeneSys; Syngene, UK), and the number with an area greater than 20 pixels were counted using image J [44]. Three scent-mark tests were performed for each subject over a period of 9 days. A consistent pattern was found within all pairs (electronic supplementary material, figure S1), and the male depositing the most scent marks in all three tests was assigned as dominant. Further analysis shows there was no difference in the number of scent marks deposited between the dominant and control males (test 1: *v* = 20, *p* = 0.84; test 2: *v* = 25, *p* = 0.38; test 3: *v* = 24, *p* = 0.46), but control males deposited significantly more scent marks than subordinate males (test 1: *v* = 28, *p* = 0.022; test 2: *v* = 19, *p* = 0.093; test 3: *v* = 21, *p* = 0.036).

To validate assignment of social status based on scent marking behaviour, preputial gland mass was also recorded. Preputial glands secrete olfactory cues into the urine of house mice, and are known to be larger in dominant males than subordinates [45,46]. As expected, males assigned a dominant social status based on scent marking behaviour had significantly larger preputial glands than their cage partner (*p* = 0.02; electronic supplementary material, table S1). By comparison, the mean preputial gland mass of control males was intermediate between that of dominant and subordinate males, although we were unable to detect significant differences between control and dominant (*p* = 0.085; electronic supplementary material, table S1) or subordinate males (*p* = 0.12; electronic supplementary material, table S1).

### (c) Measuring reproductive traits

Subjects were killed humanely at age two months when sexual maturity had been reached and a clear dominance relationship had been established within experimental pairs. Data were collected in a randomized order, with the experimenter blind to male social status. Within 30 min post-mortem, the testes, preputial glands and right seminal vesicle of each male were weighed individually and frozen whole at −20°C. The left seminal vesicle of each male was squeezed and the contents were frozen for further analysis. Epididymal sperm were isolated by macerating the right cauda epididymis on a plastic Petri dish in 100 µl of 1% citrate solution. After 2 min, a further 900 µl of 1% citrate were added and a pipette was used to homogenize and collect the sperm suspension. Sperm were counted according to standard protocols using an Improved Neubauer haemocytometer [47]. Briefly, 10 µl of the sperm suspension were added to each side of the haemocytometer. This was left in a humid box for 15 min, before counts were performed using a Leica DM1000 light microscope.

### (d) Proteomic analysis of seminal vesicle contents

The seminal vesicle secretion is a highly viscous, protein-rich substance. Defrosted samples of the secretion were weighed, diluted to a protein content of 50 mg ml^−1^ with 50 mM ammonium bicarbonate, and triturated to homogeneity. A Coomassie plus protein assay was then performed on each homogenate to accurately measure the protein concentration of each sample. Using a standard protocol, 100 µg of protein within a total final volume of 200 µl were digested using trypsin. Briefly, proteins were denatured by RapiGest SF Surfactant (Waters) at 80°C for 5 min, to assist with enzymatic digestion. The disulfide bonds in the sample were reduced and then alkylated by incubation with dithiothreitol (60°C, 10 min) followed by iodoacetamide (RT 60 min in the dark). Trypsin (0.2 mg ml^−1^, Sigma-Aldrich) was added and the sample incubated overnight at 37°C. After 12 h, 1 M hydrochloric acid and additional trypsin (0.1 mg ml^−1^) were added and left to incubate for a further 4 h to ensure complete digestion. At the end of the digestion, each sample was incubated at 37°C with trifluoroacetic acid at a final concentration of 0.5% (v/v) for 45 min. These samples were centrifuged at 17 000*g* and 4°C for 90 min and the supernatant decanted into ‘low–bind’ Eppendorf tubes. The digests were further centrifuged at 17 000*g* and 4°C for a further 90 min, and 10 µl of each digest were checked using SDS–PAGE to ensure completeness of digestion.

Seminal vesicle secretions were analysed by global proteomics using high-resolution mass spectrometry. The seminal vesicle secretion digestion mixtures were diluted 300 fold with 97 : 3:0.1 HPLC grade water : MeOH : TFA. The sample was diluted owing to the low complexity and broad dynamic range of this sample, with eight proteins accounting for over 90% of the total protein content. The tryptic peptides were resolved over a 50 min linear organic gradient of 3–40% buffer B (0.1% formic acid in acetonitrile), using a nanoACQUITY (Waters, Wilmslow, UK) ultra-performance liquid chromatography system. The HPLC system was coupled to an electrospray ionization source and an LTQ Orbitrap Velos mass spectrometer (Thermo Fisher), acquiring high-resolution mass data in a data-dependent manner. The top 20 most intense peptides in each MS scan were selected for MSMS analysis.

Progenesis LC-MS software (Nonlinear Dynamics/Waters) was used to analyse the raw HPLC-MSMS data and provide label-free relative protein abundances. This software aligns raw data from the HPLC-MSMS runs according to retention time and *m/z* values. After all peptide ions were matched, those with charge states between [M + 2H]^2+^ and [M + 4H]^4+^ were included in an aggregate file (.mgf file) that was searched using a local Mascot server (v. 2.3.01) against a protein database of reviewed UniProt *Mus musculus* entries with additional unreviewed ejaculate-specific entries of proteins identified elsewhere [30]. Mascot search parameters were set at 10 ppm peptide tolerance and 0.5 Da MSMS tolerance, with one missed tryptic cleavage, a fixed cysteinyl carbamidomethylation and variable oxidation of methionine modification. The Mascot search results were imported into Progenesis as an .xml file and protein identifications assigned to each peptide peak. Proteins with at least two unique peptides were quantified by comparing summed ion intensities for each peptide within each individual sample. Finally, Progenesis normalizes between individual LC-MSMS runs to compensate for small variances in, for example, sample loading.

### (e) Data analysis

Data analysis was performed in R (v. 3.1.0) [48]. Data were transformed as appropriate prior to analysis (detailed below). Non-parametric methods were used for the scent mark counts as the data were not normally distributed. Paired *t*-tests were performed to compare traits of dominant and subordinate males within each pair. To model the data for comparison of the dominant and subordinate males to control males, generalized linear mixed models were performed using the lme4 package in R [49] to include ‘litter’ as a random effect.

Accurate absolute quantification of proteins was not possible here but relative quantification, using the same peptide ions, was used to compare expression of the same protein in the three groups. The proteomics results, therefore, consist of compositional data with a high number of predictor variables and a low *n* number. Random forest (RF) is a robust non-parametric method of data analysis suited to analysing high-dimensional proteomics data [30,50]. Here, RF analysis was performed on samples from the control, dominant and subordinate males, and trained to classify the data according to these three groups. Data were uploaded to R (RStudio Version 1.2.5033), using the ‘compositions’ package [51], and abundance data were centred log-ratio transformed. The ‘party’ package [52] was used to perform conditional RFs (cforests) on the transformed data, to predict the classification of each individual as dominant, subordinate or control. The average accuracy was taken from 10 models, each with 1000 trees and an mtry of 5, and used to create a confusion matrix. The variable importance measures were computed using the varimp function within the party package for each of the 10 cforest models. Principal component analysis (pca) was carried out on the transformed data and linear discriminant analysis was then performed on the top 15 pca components using the MASS package [53].

## 3. Results

### (a) Ejaculate production

**Figure 1.**
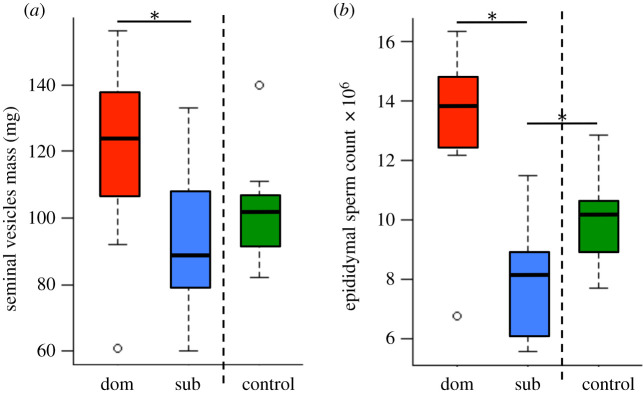
Reproductive traits and social status. Plots of male reproductive traits in relation to male social status (dominant (dom), subordinate (sub) or control): (*a*) seminal vesicles mass and (*b*) epididymal sperm count (*p < 0.05). Bars represent median and interquartile ranges. (Online version in colour.)

There was no significant difference in the body mass, testes mass or epididymides mass of dominant, subordinate and control males (electronic supplementary material, table S1). Dominant males had significantly larger seminal vesicles than subordinate and control males (electronic supplementary material, table S1; figure 1*a*), and held more sperm within their epididymides (electronic supplementary material, table S1; figure 1*b*). The same patterns were found when taking into account variation in body mass; that is, no difference in relative testes mass or epididymides mass between treatment groups, but larger seminal vesicles relative to body mass in dominant males (electronic supplementary material, table S1). In addition, subordinate males had relatively smaller seminal vesicles than control males (*p* = 0.038; electronic supplementary material, table S1). Subordinate males also had lower sperm counts than control males (electronic supplementary material, table S1; figure 1*b*). However, the seminal vesicle secretions of subordinate males had a significantly higher protein concentration than those of dominant males (d.f. = 7, *t* = −3.96, *p* = 0.005) and control males (d.f. = 7, *t* = −2.19, *p* = 0.046).

### (b) Proteomic analysis of proteins of seminal vesicle secretion

**Figure 2.**
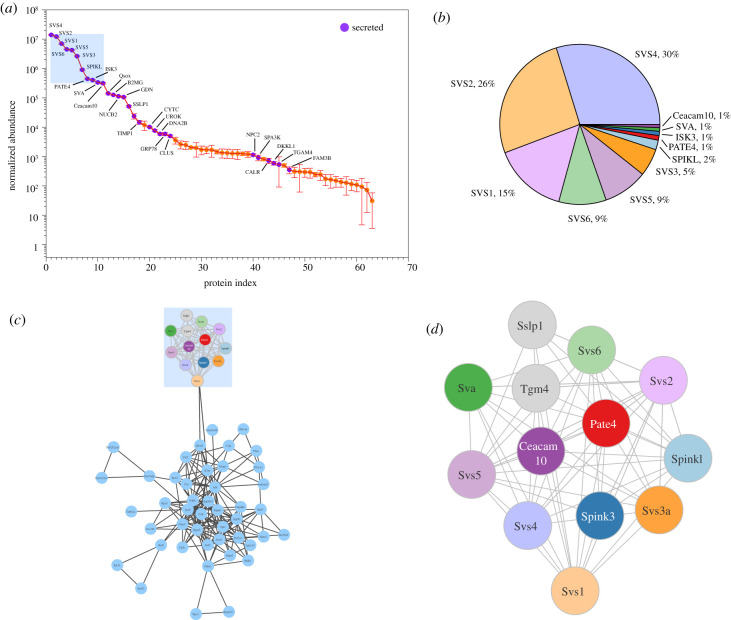
Proteomic profiling of seminal vesicle secretion. Seminal vesicle secretions were analysed by trypsin digestion, followed by high-resolution LC-MS/MS. All analyses from control, dominant and subordinate samples were combined (*n* = 24). The proteome data were analysed by label-free quantification, based on a minimum of two unique peptides for identification and a label-free approach to quantification. Proteins with signal peptides are highlighted purple. (*a*) Overall profile of protein expression, (*b*) a StringDB analysis of the proteins in (*a*) revealing two clusters, known seminal vesicle secretion contents (top) and a second cluster of low abundance, largely intracellular proteins. The true seminal vesicle secretion proteins, highlighted in (*d*), were substantially the most abundant in the samples (*c*). For protein abbreviations, see electronic supplementary material, Secreted Proteins.

**Figure 3.**
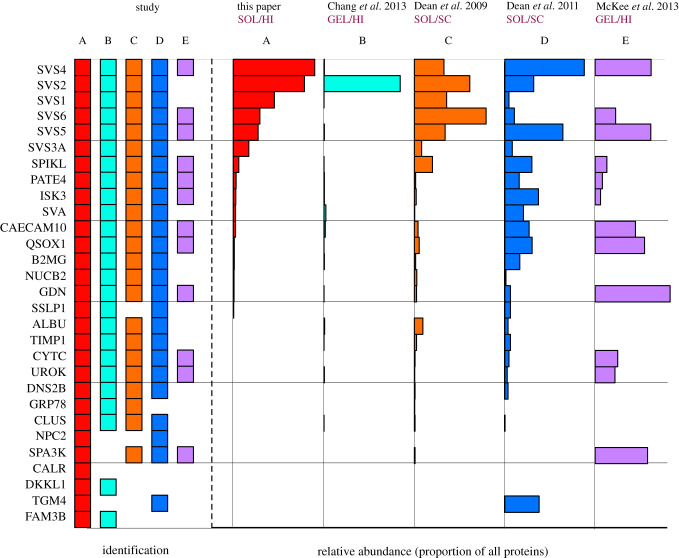
Summary of proteome studies in mouse seminal vesicle fluid. Data were compiled from a range of studies on the mouse seminal vesicle proteome. The selected proteins are based on this study (which also defined the list of true secreted proteins) as a framework on which to capture relative quantification data from the four other studies. The proteomics strategies adopted in these studies are summarized in the figure (‘GEL’, in-gel digestion from SDS–PAGE; ‘SOL’, solution digest of seminal vesicle fluid or recovered ejaculate; ‘HI’, label-free quantification based on summed peptide intensities; ‘SC’, quantification based on the number of peptides measured for each protein). Because the protein abundances cover a wide dynamic range, the key on the left-hand side of the figure indicates whether a protein was identified, regardless of quantification. For protein abbreviations, see electronic supplementary material, Secreted Proteins. (Online version in colour.)

The seminal vesicle secretion is dominated by relatively few proteins when visualized on SDS–PAGE (see below, §3c) and the instrument loading and subsequent label-free quantitative analysis were directed to obtain quantitative data on these more abundant proteins. The second group of lower abundance proteins were more heterogeneous, comprising a mixture of intracellular and secreted proteins. The non-secreted proteins are likely to contain cellular debris and are probably not true components of the seminal vesicle secretion. Across all 24 replicates, 64 proteins were confidently identified and quantified with at least two unique peptides. The protein abundances, averaged across all samples, spanned a broad range, covering at least seven orders of magnitude (figure 2*a*). However, the abundance distribution was highly biased to a few proteins that dominated the profile; the top 11 proteins were responsible for 97% of the summed label-free abundance (figure 2*b*). The protein identities were also used to explore functional categorization by known protein : protein interactions, using the StringDB tool ([54]; figure 2*c*). Two distinct groupings were clearly evident from this analysis—a tightly grouped set of proteins that mapped to all of the seminal vesicle secretion proteins SVS1–SVS6, seminal vesicle antigen (SVA), PATE4 and a number of serine protease inhibitors (figure 2*d*). This tightly linked cluster were all located in the high abundance category (figure 2*a*,*b*) and were all true secreted proteins. There have been several proteomic studies of seminal vesicle fluid in the mouse [55–58], and our list, specifically of secreted proteins, allowed us to cross-reference and attempt to compile a consensus of the abundant proteins in this fluid (figure 3). Perhaps unsurprisingly, proteins were variably present in different analyses, reflecting in part the depth to which different analytical approaches reach. In addition, some analyses were based on recovery from extruded seminal vesicle fluid, another from females post-insemination (using stable isotope labelling to discriminate male-derived from female-derived proteins [57]). In terms of the analytical approach, some studies used in-gel digestion of gel slices after SDS–PAGE fractionation [56,58], which can elicit variable results owing to uneven recovery and leaching of proteins from the gel. Other studies perform a tryptic digestion on seminal vesicle fluid extruded from the gland, as in this study. These bottom-up proteomics strategies, as might be expected for studies spanning about a decade, are thus based on different instrumentation and quantification methods and figure 3 must, therefore, be seen as a very inadequate attempt to address emergent knowledge on the mouse seminal fluid proteome. Notwithstanding such complications, a consensus in protein identifications emerges. As expected, the greatest consistency was obtained from the highest abundance proteins; these are also the proteins that have the highest abundance in figure 2*a*,*b*. There is substantial overlap between this protein list and the compilation of 69 true male-derived proteins compiled by Dean *et al.* [57], although those samples were recovered from ejaculates within the female and thus included proteins derived from the prostate, for example. To compare quantitative data, albeit with a degree of approximation, we normalized the abundance of each protein to the total abundance of the proteins in the list. These data were rather variable, obviating detailed comparison. The best correlation was obtained with the 2009 study by Dean *et al*. [55], which, in common with our study, used solution phase digestion and high-resolution proteomics (*r*^2^ = 0.57, *p* < 0.0001). These comparisons serve to emphasize that we do not possess a comprehensive or authoritative profile of the true protein complement of seminal vesicle fluid. Ideally, this would be the consensus of several different laboratories, based on a target protein list that is used to specify a group of stable isotope-labelled standards that could be shared and which would yield absolute values, for example, in terms of the number of molecules of each protein per microlitre of seminal vesicle fluid. Such absolute values would inform the stoichiometry of protease : antiprotease interactions, for example, or allow monitoring of the loss of proteins during the copulatory process (whether by physical loss or by cross-linking or degradation). Such a rigorous evaluation would have considerable potential, and generate the required tools, for a broad range of further studies.

### (c) The effect of social status on proteins of seminal vesicle secretion

Seminal vesicle secretion proteins from the three subject groups (dominant, subordinate, control) were analysed for comparative proteome expression. The 24 LC-MS/MS analyses aligned extremely well and the normalization factors (to correct for small variations in sample preparation and loading) were also very slight with chromatogram alignment scores ranging from 90 to 95% and quantitative normalization scores between 0.65 and 1.5 across all 24 runs.

**Figure 4.**
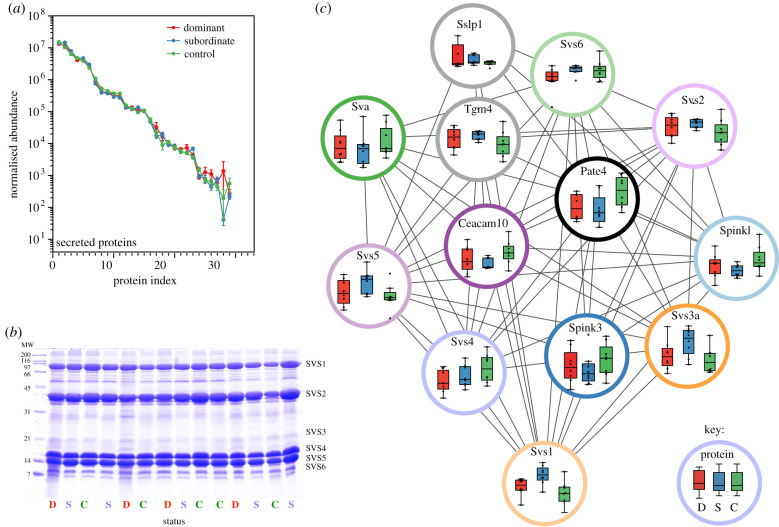
(*a*) Quantitative analysis of proteins in seminal vesicle secretion. True secreted proteins were compared quantitatively by label-free proteomics for control (C), dominant (D) and subordinate (S) groups. (*b*) To provide a different view of the protein complement of the seminal vesicle secretion, proteins were profiled by SDS–PAGE—major seminal vesicle proteins are labelled. (*c*) Additionally, label-free abundances of the proteins within the seminal vesicle cluster (figure 2) were mapped on to individual proteins (box and whiskers, median and interquartile ranges, as well as individual data displayed). The colours of the enclosing circles map to the same colours in figure 2, but the colour fill has been removed to aid clarity. For protein abbreviations, see electronic supplementary material, Secreted Proteins.

Using the aligned, normalized data, there were no substantial changes in the expression levels of any of the secreted proteins; changes in the intracellular proteins probably reflect different degrees of contamination with cells or cellular debris. Accordingly, all further analyses focused on proteins that are either known to be secreted or that identify a clear signal peptide using the SignalP server [59]. This reduced the protein list to 29 candidates and resonates well with observations from other studies (figure 3) [56]. The secreted proteins are largely the most abundant (figure 2), spanning between four and five orders of magnitude in label-free abundance. Further, the label-free abundance of each protein did not vary considerably between dominant, subordinate or control groups (figure 4*a*). This is confirmed by the consistency of SDS–PAGE analysis of the seminal vesicle fluids (figure 4*b*), highlighting a reduced set of major bands corresponding to those at the top of the label-free abundance list. For the individual proteins in the ‘seminal vesicle’ cluster, the individual label-free abundances for the proteins in the three groups were extracted and plotted individually, and emphasize the subtlety of the changes (figure 4*c*).

**Figure 5.**
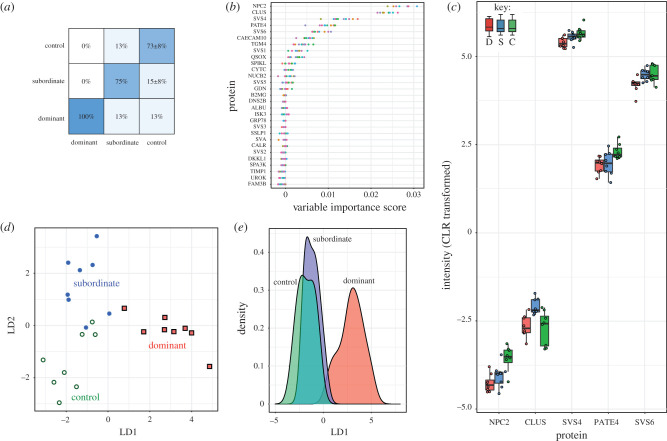
Multivariate comparison of the seminal vesicle proteins. Seminal vesicle secreted proteins were centre log transformed before the data were analysed using RF analysis. The confusion matrix (*a*) indicates the quality of the classification, and the variable importance scores (*b*) rank the proteins according to their usage and strength in tree construction over 10 sets of 1000 trees (each point for any single protein is one set of 1000 trees). Each set of trees is discriminated with a distinct colour. The abundances of the five proteins with the highest scores in (*b*) are plotted in (*c*). The same data were also used for principal component analysis, and the 15 most significant principal components were used in a linear discriminant analysis (*d*,*e*). For protein abbreviations, see electronic supplementary material, Secreted Proteins.

Although in classical proteomics terms, the observed changes in seminal vesicle fluid proteins appear to be small, biology rarely operates on large-fold differences and it is possible that a subtle adjustment of the overall seminal vesicle fluid composition could be as effective in yielding a consistent biological response. Accordingly, the secreted protein abundance data were analysed using RF analysis to discern more subtle changes in the protein profile. The dataset, of 29 proteins and 24 cases (three groups of eight), was used to generate 10 sets of 1000 trees. From these analyses, the overall performance in terms of discrimination of dominant and subordinate was reasonable (figure 5*a*), achieving 84% accuracy that was essentially stable (±1%) for 10, 100 or 1000 iterations, each comprising 1000 trees. From the 10 iteration analysis, the variable importance scores of the different variables were highly consistent, with Niemann-Pick Protein 2 (figure 5*b*, NPC2), clusterin (CLUS), SVS4, prostate and testis expressed protein 4 (PATE4) and SVS6 ranking the highest. The abundances of these five proteins are plotted in figure 5*c*. Some proteins, such as NPC2, were effective in resolving control animals from either subordinate or dominant, while other proteins, such as clusterin and SVS6, differed between subordinate and dominant mice. This was confirmed by linear discriminant analysis of the top 15 PCA components (figure 5*d*), where the first linear discriminant component was highly effective at resolving dominant samples from the other two categories. However, comparisons of the abundances of individual proteins did not reveal major changes. To illustrate, a paired *t*-test comparing dominant and subordinate males for SVS5 (*t* = 4.16, *p* = 0.004, d.f. = 7), clusterin (*t* = 2.75, *p* = 0.029, d.f. = 7) and CYTC (*t* = −2.44, *p* = 0.045, d.f. = 7) yielded individual *p*-values < 0.05, but none pass corrections for multiple comparisons (see electronic supplementary material).

## 4. Discussion

We find evidence that both sperm and seminal fluid protein investment differ according to social status in male house mice. Dominant males invested more in ejaculates overall, with significantly higher sperm counts and larger seminal vesicles than subordinate and control males. However, subordinate males produced a more concentrated protein secretion from their seminal vesicles, and we found subtle but consistent differences in the seminal fluid protein composition of male mice according to their social status. These differences in the composition of the seminal vesicle secretion highlight that comparing gross production measures, such as gland size, may mask differences in the relative production of specific proteins that could have functional significance to a male's fertilization success. Similarly, evidence for differences in sperm production according to social status were found, despite no significant difference in testes mass (see also [60]).

Subordinate male mice showed a reduced overall investment in ejaculates, with lower epididymal sperm counts and smaller seminal vesicles than dominant males, despite having similar body masses. Decreased investment in sperm by subordinate males has previously been reported for laboratory mice [24,41] and bank voles (*Myodes glareolus* [26]). Similar to the findings reported here, subordinate male bank voles also have smaller seminal vesicle glands than dominants [26].

A comparison of the reproductive traits of subordinate males in our study with those of singly housed males suggests that some aspects of subordinate males' reproductive function is being suppressed in the presence of a dominant male. This is also consistent with the dichotomous pattern of scent marking typical of dominant and subordinate male mice [43], and may reflect lowered testosterone levels of subordinate males [25]. As discussed by Lemaitre *et al.* [26], social suppression of reproductive function under competitive conditions could impose significant constraints on ejaculate investment decisions. This may explain why subordinate male rodents do not invest more in sperm production, as predicted by sperm competition theory (e.g. [8]), whereas males in other taxa have been shown to do so when mating in a disadvantaged mating role (e.g. [23,61,62]). Conversely, the expression of some reproductive traits is heightened in dominant males compared to singly housed males, suggesting that an increased reproductive investment by dominant males may be stimulated by direct competition. However, considering the seminal vesicle secretion of subordinate male mice, we found a higher protein concentration in the secretion compared to dominants. Hence, it is possible that some partial compensation for reduced sperm output by subordinate males might be afforded by adjustments to the amount and/or composition of seminal vesicle proteins, despite their overall lower investment.

Seminal fluid components are known to have important effects on the outcome of postcopulatory sexual selection (e.g. [63,64]), and may, therefore, be adjusted according to mating roles under sperm competition risk. For example, recent studies demonstrate that males can plastically alter the production and secretion of specific seminal fluid proteins with functional significance in postcopulatory sexual selection according to local conditions [12,13,16,65–67]. Here, although it is clear from multiple analyses (SDS–PAGE, label-free proteomics) that there is no dramatic change in protein composition, a combination of modest changes in several proteins could still potentially alter ejaculate composition to enhance sperm competition success according to social status. The seminal vesicle secretion contains several classes of proteins; the coagulum proteins SVS1–6, protease inhibitors (SPIKL, ISK3, GDN) and nucleases (DNS2, NUCB2), all of which act to influence fertilization success, through optimization of coagulation plug stability, suppression of proteases secreted by the female into the reproductive tract and hydrolysis of neutrophil NETS, webs of DNA that contain multiple bound proteins, including proteases [68]. Small adjustments to several of these proteins might, therefore, combine to elicit an advantage according to mating role, which resonates well with the known or proposed functions of key proteins that allowed discrimination according to social status in our dataset. Moreover, among proteins with the top 10 highest variable importance scores in our RF analysis, six (SVS1, SVS4, SVS6, SVS7, Caecam10 and SPIKL) have previously been suggested to change plastically in response to sperm competition risk [28]. Among these, SVS6 has been suggested to function as a protease inhibitor [69] and could, for example, protect the mating plug against liquefaction in the female reproductive tract. Expression of SVS6 for males in our study was relatively high both for subordinate and singly housed males compared to dominant males. Hence, it appears that dominant males have reduced investment in SVS6, which might reflect a lower risk of their mating plug being prematurely displaced or ejected. By contrast, clusterin was elevated in subordinate males relative to both dominants and controls. Clusterin is an extracellular chaperone that functions to prevent stress-induced aggregation of high concentrations of protein or its selective uptake [70,71]. It might be argued that the higher protein concentration of clusterin in subordinate seminal vesicle fluid could align with the higher total protein concentration, which would be a stimulus for increased aggregation. In RF analysis, the highest variable importance score was for Niemann-Pick Protein 2, a sterol-binding protein that is involved in sperm maturation in the epididymis [72]. This protein is also secreted by seminal vesicles, but its role in ejaculates is unclear [73], as is the observation of lower levels in both subordinate and dominant animals compared to controls.

Our finding that subordinate male mice appear to be investing relatively more in specific functionally significant seminal fluid proteins, as well as having an overall increased concentration of seminal fluid proteins, is consistent with certain theoretical predictions in relation to optimal investment strategies for seminal fluid components [9]. This theory predicts that for species in which seminal fluid components can significantly affect fertility, males mating in a disfavoured role may gain a greater advantage through increasing their seminal fluid output. Our results partly support this, since despite their reduced sperm output, subordinate males are producing significantly more of certain proteins compared to dominant males, and the overall composition of their secreted seminal vesicle proteins is distinct from that of dominant males. These findings are also consistent with limited evidence, suggesting that the mating plugs produced by subordinate males are comparable in size to those of dominant males [74]. Notably, the seminal fluid secretion of dominant males also appears distinct from that of both subordinate and control males, suggesting that the dominant male ejaculate may be optimized for mating in a favoured role under an elevated risk of sperm competition.

However, there remains a need for higher quality assessment of the abundances of these proteins in seminal vesicle secretion, ideally one that does not require alignment and normalization of the entire dataset. In this regard, absolute quantification rather than relative label-free quantification is required to dissect changes in the seminal vesicle secreted proteome. This would require the use of stable isotope-labelled standard peptides, several per target protein, that are used as a reference standard. Multiple proteins are readily quantified through QconCAT technology, which creates an artificial protein that is a concatamer of all peptides that are needed for quantification [75,76].

In the absence of ejaculation, seminal vesicle proteins have a turnover rate with a half time of about 10 days [77], which sets a limit on the rate of change in the protein composition that can be elicited. However, post-ejaculation, the replenishment of seminal vesicle secreted proteins would be much more sensitive to the rate of synthesis. Since subordinate males have fewer mating opportunities, they may lack the opportunity for rapid change in composition that would be afforded to dominant males that mate more frequently. It is possible that comparison of the protein profile would be more informative if assessed in the context of number of copulations to deplete and thus, replenish the seminal vesicle secretion. Further quantification would also be useful to explore how the differences in sperm and seminal fluid protein production found here translate into ejaculates recovered under different conditions [16]. Although available evidence for house mice suggests that dominant males ejaculate more sperm than subordinates under controlled conditions [74], the extent to which social status interacts with varying levels of sperm competition to influence overall ejaculate composition is unknown.

In conclusion, this study combines behavioural and proteomic techniques to show that dominant and subordinate male house mice exhibit distinct reproductive phenotypes. A novel application of emerging techniques has shown that subordinate male house mice may compensate for lower overall investment in ejaculates by increasing the concentration of proteins in their seminal fluid and increasing the production of specific seminal fluid proteins linked to mating plug function and fertility. Moreover, the composition of dominant males' seminal fluid is also distinct from that of socially isolated males, which could reflect optimization to a favoured role under elevated sperm competition risk. Overall, our findings highlight the importance of considering the entire ejaculate when studying investment strategies. This is particularly important in species that produce a copulatory plug from their seminal fluid, as differential investment in specific proteins could greatly affect fertilization success.

## Supplementary Material

Supplementary Data Secreted Proteins

## Supplementary Material

Supplementary Data Reproductive Traits

## Supplementary Material

Supplementary Figure S1 and Table S1

## Data accessibility

This publication is supported by multiple datasets. The proteomics data have been deposited to the ProteomeXchange Consortium via the PRIDE partner repository with the dataset identifier PXD019727. All other data are provided as electronic supplementary information.
