# Supplementary material for: Social status and ejaculate composition in the house mouse
Source: Philos Trans R Soc Lond B Biol Sci. 2020 Oct 19;375(1813):20200083. doi: 10.1098/rstb.2020.0083 (PMC7661446; doi:10.1098/rstb.2020.0083)
Supplement: Supplementary Data Secreted Proteins [file rstb20200083supp1.pdf]

Social status and ejaculate composition in the house mouse

Helen L. Bayram, Catarina Franco, Philip Brownridge, Amy J. Claydon, Natalie Koch, Jane L. Hurst, Robert J. Beynon, Paula Stockley

Supplementary information

Figure S1. Scent marks and social status

Typical scent marking patterns from (a) dominant and (b) subordinate male house mice.

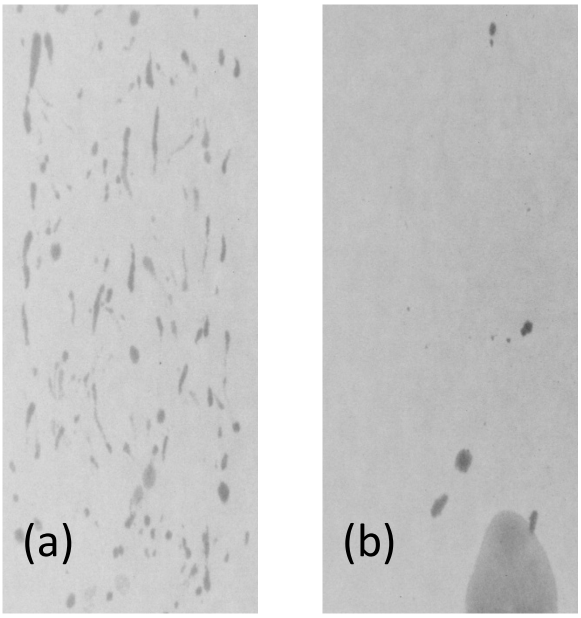

Table S1. Comparison of male reproductive traits according to social status. Differences between dominant and subordinate males are tested with paired t-tests. Comparisons of control and dominant or subordinate males are reported using mixed models with litter included as a random factor (see methods). Additional analyses test for differences in residual testes, epididymides and seminal vesicles masses, to compare results with control for variation in body mass.

Reproductive trait	Dominant		Subordinate		Control		Dominant v. Subordinate			Control v. Dominant			Control v. Subordinate		
	Mean	s.e.	Mean	s.e.	Mean	s.e.	df	*t*	*P*	df	t	*P*	df	*t*	*P*
Body mass (g)	21.81	0.98	21.24	1.09	20.74	0.77	7	0.54	0.603	15	-0.8	0.454	15	0.05	0.960
Preputial glands (mg)	90	13	56	10	70.25	16.7	7	2.93	**0.022**	15	-1.9	0.085	15	1.64	0.122
Epididymal sperm count ($x10^6$)	13.1	1.03	7.89	0.7	10	0.55	7	4.28	**0.004**	15	-4.6	**0.000**	15	3.92	**0.001**
Testes (mg)	181.6	6.23	183.4	9.69	180	5.81	7	-0.1	0.896	15	0	0.993	15	-0.3	0.754
- with control for body mass							7	-0.47	0.652	15	0.55	0.589	15	-0.51	0.620
Epididymides (mg)	62.75	1.64	60	2.66	58.88	1.91	7	0.87	0.415	15	-1.3	0.221	15	0.31	0.758
- with control for body mass							7	0.84	0.430	15	-1.07	0.301	15	0.32	0.752
Seminal vesicles (mg)	119.3	10.7	93	8.03	103	6.33	7	2.62	**0.034**	15	-2.40	**0.003**	15	1.78	0.095
- with control for body mass							7	3.71	**0.008**	15	-2.91	**0.011**	15	2.27	**0.038**
